# The renal resistive index is associated with microvascular remodeling in patients with severe obesity

**DOI:** 10.1097/HJH.0000000000003434

**Published:** 2023-04-06

**Authors:** Diego Moriconi, Alessandro Mengozzi, Emiliano Duranti, Federica Cappelli, Stefano Taddei, Monica Nannipieri, Rosa Maria Bruno, Agostino Virdis

**Affiliations:** a Department of Clinical and Experimental Medicine, University of Pisa, Pisa, Italy; b Center for Translational and Experimental Cardiology (CTEC), Department of Cardiology, University Hospital Zurich, University of Zurich, Zurich, Switzerland; c Université Paris Cité, Inserm, PARCC, Paris, France

**Keywords:** microcirculation, obesity, renal resistive index

## Abstract

**Background:**

Renal hemodynamics is impaired since the early stage of cardiometabolic disease. However, in obesity, its noninvasive ultrasound assessment still fails to provide pathophysiologic and clinical meaningfulness. We aimed to explore the relationship between peripheral microcirculation and renal hemodynamics in severe obesity.

**Methods:**

We enrolled fifty severely obese patients with an indication for bariatric referring to our outpatient clinic. Patients underwent an extensive reno-metabolic examination, paired with Doppler ultrasound and measurement of the renal resistive index (RRI). On the day of the surgery, visceral fat biopsies were collected to perform an *ex-vivo* complete microcirculatory assessment. Media-to-lumen ratio (M/L) and vascular response to acetylcholine (ACh), alone or co-incubated with N^G^-nitro arginine methyl ester (L-NAME), were measured.

**Results:**

Patients were stratified according to their normotensive (NT) or hypertensive (HT) status. HT had lower estimated glomerular filtration rate and higher RRI compared to NT, while the presence and extent of albuminuria were similar between the two groups. Concerning microcirculatory assessment, there were no differences between groups as regards the microvascular structure, while the vasorelaxation to ACh was lower in HT (*P* = 0.042). Multivariable analysis showed a relationship between M/L and RRI (*P* = 0.016, St. *β* 0.37) and between albuminuria and the inhibitory response of L-NAME to Ach vasodilation (*P* *=* 0.036, St. *β* = −0.34). Notably, all these correlations were consistent also after adjustment for confounding factors.

**Conclusions:**

The RRI and albuminuria relationship with microvascular remodeling in patients affected by severe obesity supports the clinical implementation of RRI to improve risk stratification in obesity and suggests a tight pathophysiologic connection between renal haemodynamics and microcirculatory disruption.

## INTRODUCTION

Obesity increasing prevalence [1] is deemed to fuel cardiovascular (CV) mortality in the next decade [2,3]. Indeed, the obese vascular phenotype [4] is characterized by an increase in systemic arterial stiffness [5], vascular endothelial dysfunction [6] and microvascular hypertrophic remodeling [7]. Also, obesity is associated with albuminuria [8], a marker of microvascular endothelial damage [9]. As this early vascular impairment leads to profound changes in renal hemodynamics [10], detecting the vascular status in a patient with obesity might be crucial for adequate risk stratification.

However, the noninvasive exploration of several vascular districts in patients with obesity often fails to convey clinical meaningfulness. With particular regard to the kidney, some authors suggest that the renal resistive index (RRI) might reflect the presence of arteriolosclerosis and interstitial fibrosis, which follows the development and progression of chronic kidney disease [11]. Intraparenchymal arterial waveform could be considered the result of vascular compliance and resistance, making the RRI a reliable index of downstream microvascular impedance [12]. On the other hand, some studies show that RRI is markedly influenced by systemic rather than renal hemodynamics, above all the pulsatile component of the systemic blood pressure [13] and by damage in the large arteries, that is, aortic stiffness [14,15], rather than in the microcirculation. In a recent study on normotensive patients on the waiting list for bariatric surgery, RRI correlated inversely with renal vascular resistance and directly with renal plasma flow, suggesting that, in severe obesity, RRI might be an index of hyperperfusion rather than arteriolosclerosis [16]. Thus, the clinical significance of RRI remains a matter of debate [17].

The difficult interpretation of the current evidence might partially be due to the lack of studies exploring RRI and microvascular remodeling by reliable techniques simultaneously. Indeed, many studies suggest that obesity [18], as well as other metabolic diseases [6], is characterized by microvascular rather than macrovascular alterations since their early stages [19]. Thus, to dissect the potential usefulness of RRI is mandatory to explore its correlation with microvascular structure and function.

We conceived the present study to explore the relationship between peripheral microcirculation and renal hemodynamics in severe obesity. In particular, we aimed to investigate whether an ultrasound-based assessment of renal hemodynamics (e.g. RRI) might be a proxy for systemic morphofunctional microvascular alterations and whether this relationship is independent of systemic blood pressure and other acknowledged confounders. Secondly, we investigated whether albuminuria, another marker of renal microvascular dysfunction, is associated with systemic microvascular alterations. Finally, we explored whether any of those relationships are conditioned by the concomitance of arterial hypertension (i.e. a systemic hemodynamic perturbance).

## MATERIALS AND METHODS

### Study design and population

In this cross-sectional study, the population was selected among patients affected by morbid obesity (BMI > 35 kg/m^2^) on the waiting list for bariatric surgery. We selected individuals with the following characteristics: having participated in a research protocol including the ex-vivo microvascular structure and function assessment by our laboratory between 2015 and 2019, and having performed a presurgery renal ultrasound with RRI assessment as part of their routine clinical workup. Thus, it should be noted that a large portion of the study participants has previously taken part in published studies involving microcirculatory exploration by our lab [19,20].

The exclusion criteria were: age <18 years or >80 years, the presence of chronic comorbidities (except hypertension or type 2 diabetes).

A total of *n* = 109 patients were screened among individuals whose microcirculatory structure and function were previously analysed by our lab. After the initial screening, *n* = 59 were excluded due to missing RRI. A final sample size of *n* = 50 patients was obtained.

All patients gave their informed consent to participate in the study, which was conducted in accordance with the ethical guidelines proposed by the Declarations of Helsinki and approved by the Institutional Ethics Committee (study protocol number 12589).

### Clinical visit and biochemical assessment

After an overnight fast, patients were admitted to our outpatient unit as part of their routine presurgery clinical workup (Medicine 1 Unit, Azienda Ospedaliera Universitaria Pisana). Demographic, anthropometric and clinical parameters were recorded. More specifically, height, waist, and hip circumferences were measured at the nearest cm with a flexible narrow meter. Body weight (BW) was measured with an electronic scale. Blood pressure was measured by a digital electronic manometer with a suitable cuff according to arm circumference after sitting for >10 min. Venous blood and urinary samples were collected for standard biochemistry.

### Renal resistive index measurement

Three velocimetric measurements of the interlobular arteries (superior, middle, and inferior) in the meso-renal region of each kidney were taken with a translumbar or anterior ultrasound approach (MyLab 25, Esaote). The RRI was calculated using the following formula: RRI = (peak systolic velocity – end-diastolic velocity)/peak systolic velocity, where peak systolic velocity and end-diastolic velocity were measured in the same wave. All measurements were performed by one experienced investigator. Intraobserver reproducibility of measurements was assessed; the intraclass correlation coefficient was 0.95–0.97 with an expected variability of ≤±3%.

### Kidney function assessment

Serum and urinary concentrations of creatinine were measured with Jaffé method traceable to IDMS reference method (CREA Roche/Hitachi 917; Roche Mannheim, Germany). Urinary albumin excretion was measured from an early-morning urine sample as the urine albumin:creatinine ratio (ACR). The urinary albumin concentration was determined by immunoturbidimetric assay.

### Microvascular evaluation

Biopsies of adipose tissue recovered during surgery were immediately processed to isolate small resistance arteries (150–300 μm lumen diameter) as previously described [21]. After isolation, arteries were mounted in a pressurised myograph (DMT 114P; Danish Myo Technology A/S, Hinnerup-Denmark) to assess their structural and functional characteristics. Vessels were then rested in a Krebs [22] solution at 37°C and perfused ad +60 mmHg. KCl (125 mmol/l) + norepinephrine (1 μmol/l; Sigma Aldrich SRL, Milano-Italy) were added to the solution to assess vessel viability and vitality. Minimum (*D*_m_) and maximal (*D*_M_) vessel diameters were assessed as previously described [19]. Media thickness and lumen diameter were measured at three different points from each small artery to obtain the media-lumen ratio (M/L). Media cross-sectional area (MCSA) was obtained by subtracting the internal from the external cross-sectional areas using outer plus lumen diameters, as previously described [23]. Endothelium-dependent vasorelaxation was assessed by cumulative concentrations of acetylcholine (ACh, 0.001–100 μmol/l; Sigma Aldrich SRL) in vessels precontracted with norepinephrine (1 μmol/l, Sigma Aldrich SRL). Vasodilation response was described as % changes to the maximal vessel diameter, as previously described [19]. NO availability was investigated by repeating the dose-response curves to acetylcholine after incubation with the NO synthase inhibitor N^G^-nitro arginine methyl ester (L-NAME) (100 μmol/l, 30 min-incubation, Sigma Aldrich SRL).

### Statistical analysis

Quantitative data were expressed as mean ± SD or median [interquartile range] for variables with normal or skewed distribution, respectively. Continuous variables with a normal distribution were compared between individuals with or without hypertension by the Student's *t*-test, while the variables with a skewed distribution by the Mann−Whitney *U* test. Categorical variables were analysed with the χ^2^ test. Pearson's test was used to explore correlations among variables in univariate analysis. When significant differences were found in univariate analysis, variables were further compared by ANCOVA, adding age, sex, HbA1c, MBP and PP as covariates.

Statistical tests were performed using JMP Pro 16.3.0 (SAS Institute Inc., Cary, North Carolina, USA) using a two-sided α level of 0.05.

## RESULTS

### Anthropometric and biochemical features

The main anthropometric and biochemical characteristics of the study participants are summarized in Table 1. As hypertension was highly prevalent in our cohort (42%), the patients were divided into two groups based on the presence (HT) or absence (NT) of hypertension to discern the obesity-specific relevance of RRI. Age was significantly higher in the HT group, whereas sex distribution, BMI and smoking habit were similar between the two groups. The HT group had a higher prevalence of type 2 diabetes mellitus; consequently, the HT group also had higher levels of fasting glucose and HbA1c compared to the NT group.

**TABLE 1:** Anthropometrics and clinical features of participants affected by severe obesity

	NT	HT	*P* value	*P*_adj_ value
*n*	29	21		
Age (years)	38 ± 12	55 ± 8	<0.0001	<0.0001
Sex (m/f)	6/23	8/13	ns	–
Type 2 diabetes (y/n)	1/28	8/13	0.002	0.001
Albuminuria (y/n)	4/25	4/16	ns	–
Smoking habit	5/24	4/17	ns	–
BMI (kg/m^2^)	42.3 ± 7.9	43.2 ± 5.2	ns	–
SBP (mmHg)	125 ± 10	133 ± 7	0.003	0.006
DBP (mmHg)	79 ± 8	78 ± 9	ns	–
PP (mmHg)	45 ± 9	50 ± 5	0.028	ns
MBP (mmHg)	95 ± 7	99 ± 6	0.019	0.017
Heart rate (bpm)	85 ± 11	88 ± 12	ns	–
Glycaemia (mg/dl)	96 ± 11	102 ± 10	0.029	0.039
HbA1c (mmol/mol)	37 ± 4	42 ± 9	0.038	0.004
Total cholesterol (mg/dl)	187 ± 32	184 ± 35	ns	–
HDL (mg/dl)	47 ± 13	40 ± 10	ns	–
LDL (mg/dl)	112 ± 24	110 ± 35	ns	–
Triglycerides (mg/dl)	137 ± 61	156 ± 62	ns	–
AST (mg/dl)	28 ± 7	27 ± 8	ns	–
ALT (mg/dl)	27 ± 9	27 ± 12	ns	–
Uric acid (mg/dl)	6.1 ± 1.5	6.2 ± 1.2	ns	–
eGFR (ml/min per 1.73 m^2^)	111 ± 20	89 ± 21	0.001	<0.0001
ACR (mg/g)	13 [7–20]	12 [8–20]	ns	–
CRP (mg/dl)	8.1 ± 6.8	9.6 ± 5.9	ns	–

By definition, the HT group had higher SBP, PP and MBP, while no difference was shown in DBP and HR between groups. The mean estimated glomerular filtration rate (eGFR) was significantly lower in the HT group, while the presence and the extent of albuminuria were similar, with the same prevalence of albuminuric patients in both groups.

Finally, there were no differences in lipid and hepatic profile, uric acid and inflammatory markers between the two groups (Table 1). Notably, all these differences between HT and NT groups remained still significant after adjustment for sex (Table 1).

### Renal resistive index and ex-vivo microcirculatory function and structure

Concerning renal ultrasound, there was no difference in longitudinal renal diameter between groups, while the HT group had significantly higher values of RRI (0.66 ± 0.03 vs. 0.63 ± 0.04, HT vs. no-HT, respectively, *P* = 0.002).

**FIGURE 1:**
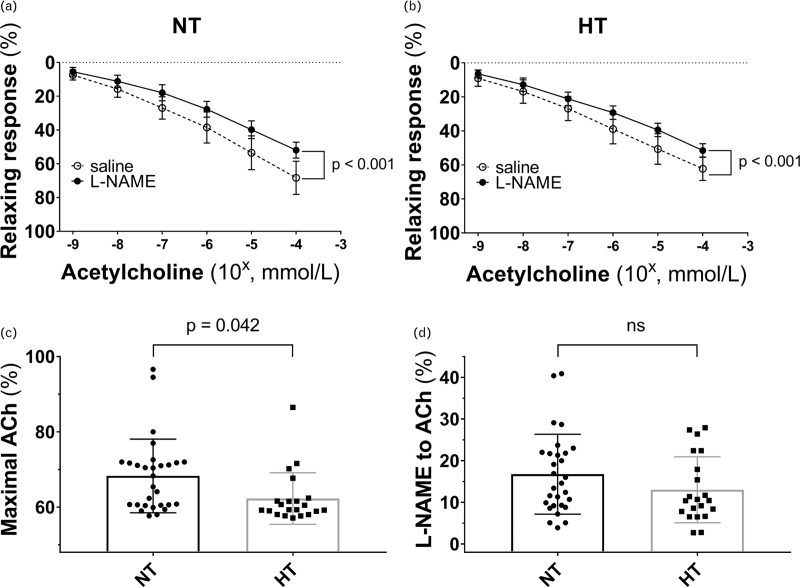
(a, b) Relaxing response to cumulative concentration to ACh in vessels precontracted with norepinephrine in the two groups. Vasodilatory response is expressed as a percentage of the maximal diameter. The experiment was repeated two times for each patient by incubating the vessel with saline (white circle, dotted line), L-NAME (black circle, continuous line). (c) Maximal vasodilatory response to Ach expressed as a percentage of the maximal diameter in the two groups (patients with obesity without hypertension: black circles, black bars; patients with obesity and hypertension: black square, grey bar) in vessel precontracted with norepinephrine. (d) Maximal inhibitory response to L-NAME expressed as a percentage of the maximal diameter in the two groups (obese without hypertension: black circles, black bars; obese with hypertension: black square, grey bar) in vessel precontracted with norepinephrine. Data are presented as mean ± SEM and were compared by ANOVA for repeated measures. Maximal vasodilatory response to ACh (ΔACh) and maximal inhibitory response to L-NAME in the two groups were compared by independent samples Student *t*-test. A *P*-value <0.05 was considered significant. ACh, acetylcholine; HT, patients with obesity and hypertension; NT, patients with obesity without hypertension.

Concerning microcirculatory assessment (Fig.1, Table 2), there were no differences between groups as regards the arteriolar diameter, resting lumen, media thickness, media-to-lumen ratio, and MCSA. The vasorelaxation to ACh was lower in the HT group (64.5 ± 8.0% vs. 68.5 ± 9.6%, HT vs. NT, respectively, *P* = 0.042). In contrast, no differences between groups were shown in vasorelaxation after the inhibition with L-NAME.

**TABLE 2:** Renal diameter, renal resistive index and ex-vivo microcirculatory function and structure

	NT	HT	*P* value	*P*_adj_ value
*n*	29	21		
Renal resistive index (RRI)	0.63 ± 0.04	0.66 ± 0.03	0.021	0.036
Renal longitudinal diameter (cm)	11.9 ± 0.9	11.8 ± 0.8	ns	–
Resting lumen (μm)	225.2 ± 23.2	216.2 ± 15.3	ns	–
Media thickness (μm)	21.4 ± 4.0	21.5 ± 4.0	ns	–
M/L ratio	0.09 ± 0.02	0.10 ± 0.02	ns	–
MCSA (μm^2^)	16735 ± 4534	16250 ± 3870	ns	–
Maximal vasodilation to Ach (%)	68.5 ± 9.6	64.5 ± 8.0	0.042	ns
Maximal vasodilation to Ach + L-NAME (%)	51.9 ± 4.6	51.7 ± 4.0	ns	–
L-NAME to ACh (%)	16.7 ± 9.5	13.0 ± 7.9	ns	–

In gender-adjusted analysis RRI remained significantly higher in HT group compared to NT group (*P*_adj_ = 0.036, Table 1), while there were no more statistically significant differences were found in vasorelaxation to ACh between groups.

### Regression analysis

In univariate analysis, the RRI was positively correlated with age (*r* = 0.36, *P* = 0.013), SBP (*r* = 0.32, *P* = 0.011), PP (*r* = 0.31, *P* = 0.017) and media-to-lumen ratio (*r* = 0.39, *P* = 0.003); RRI and the inhibitory response of L-NAME to ACh vasodilation showed a positive trend (*r* = 0.25, *P* = 0.059). No correlation was found between RRI and BMI, MBP, as well as parameters of kidney function such as albuminuria and eGFR, and the other microcirculatory parameters (MCSA, vasorelaxation with ACh).

Albuminuria was positively correlated with DBP (*r* = 0.31, *P* = 0.024), MBP (*r* = 0.29, *P* = 0.029) and negatively with the inhibitory response of L-NAME to ACh vasodilation, a direct measure of endothelial function (*r* = 0.30, *P* = 0.036). No correlations were found between albuminuria and age, BMI, SBP, PP, eGFR, and structural parameters of microcirculation such as media-to-lumen ratio and MCSA.

**FIGURE 2:**
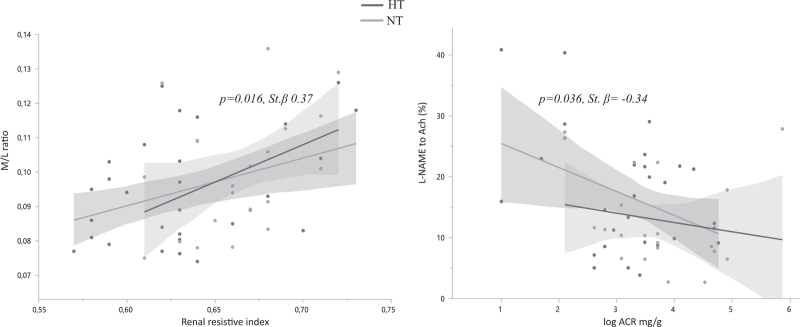
Multiple regression analyses. (a) RRI correlation with M/L in HT and NT patients (*P* = 0.001, St. *β* = 0.41). (b) Correlation between albuminuria and the inhibitory response of L-NAME to Ach vasodilation (*P* = 0.036, St. *β* = *−*0.34). Independent variables: age, sex, MBP, HbA1c. ACh, cetylcholine; HbA1c, glycated hemoglobin; L-NAME, L-N^G^-nitro arginine methyl ester; MBP, mean blood pressure; RRI, renal resistive index.

Multivariable analyses were run to investigate whether the association between microvascular and renal parameters were independent of common confounders. M/L was associated with RRI (*P* = 0.001, St. *β* = 0.41), independent of confounding factors such as age, sex, MBP and HbA1c. This association was confirmed either in NT or in HT (Fig. 2a). Another significant association was found between albuminuria and the inhibitory response of L-NAME to ACh vasodilation in the presence of the same confounders (*P* = 0.036, *St. β =* −0.34). This association, too, was confirmed either in NT or in HT. (Fig. 2b).

We repeated those analyses by replacing MBP with PP in the model to verify the impact of pulsatile rather than the steady component of systemic pressure. RRI remained significantly associated with M/L (*P* = 0.026, St. *β* = 0.37); similarly, albuminuria was confirmed to be independently associated with L-NAME-to-Ach vasorelaxation (*P* = 0.031, St. *β* = −0.47).

## DISCUSSION

The main findings of the present study are: in severe obesity, renal resistive index is positively associated with structural microvascular remodeling, described by media-to-lumen ratio; this association is independent of mean blood pressure and hypertension status; in patients with severe obesity higher levels of albuminuria are associated with impaired inhibition of L-NAME to ACh vasodilation, a direct measure of endothelial function, independently of the hypertensive status. Taken as a whole, our findings provide a novel pathophysiological insight into RRI.

Most studies found a significant and independent association of RRI value with age and systemic blood pressure [24,25], which we confirmed in our obese population. Furthermore, we found a positive linear correlation between RRI, SBP and PP. This latter association had already been demonstrated in previous studies [13,26], suggesting that the pulsatile component of blood pressure affects RRI values. However, in a multiple regression model including microvascular structure and function variables, this association was no longer significant. At the same time, we reported a strong positive correlation between RRI and structural microvascular remodeling, assessed with the media-to-lumen ratio. A possible explanation arises from the evidence that in severe obesity, small resistance arteries suffer from hypertrophic remodelling since the early stages [27]. Obesity is an acknowledged model of microvascular ageing. In previous studies from our group [19,20], we reported that the curve of M/L plotted against age from obese patients and healthy control diverges starting from an early age (about 20 years), conceivably before hypertension onset. This remodeling could evolve independently of the inward eutrophic remodeling typically associated with the first stages of arterial hypertension [28]. It could be driven by interaction among local growth factors, inflammatory cytokines and oxidative stress [4,29]. In fact, in an obese murine model, Ortega *et al.*[30] showed that vascular outward remodeling was associated with adipokine dysregulation and it is well known that severe obesity is characterized by constant stimulation of inflammatory cascade promoted by adipose tissue that chronically induces vascular fibrosis in humans [3,6,31]. It should also be noted that we did not find a significant relationship between MCSA and RRI. However, although MCSA is a relevant parameter to assess vascular structure, there is broad consensus [32] that myography-assessed M/L has proven to be particularly effective in discriminating between phenotypes at different cardiovascular risk, as consistent evidence from different groups demonstrates [33–35]. Also, our findings agree with what was observed in other observations assessing the correlation between microvascular indices at different body districts, such as between the subcutaneous and the retinal one [36]. M/L is independent of vessel dimensions and thus is considered the best indicator for small vessels’ morphology assessed through myography [36,37]. Similarly, RRI is independent as well from the vessels’ size. Thus, it is reasonable to observe a better agreement between these two measures.

Under the hypothesis that these changes progress in parallel in different organs (including the adipose tissues and the kidney), the progressive high-impedance microvascular ambient deriving from this structural remodeling would be the main factor associated with the simultaneous increase in the RRI in severe obesity. This could be independent of the analogous changes that hypertension induces at the microvascular level.

Higher RRI has been associated with subclinical signs of kidney damage in lean patients with primary hypertension without CKD, demonstrating a direct link with albuminuria [38], and a study conducted on hypertensive individuals showed that each 0.1 increase in RRI was associated with a 5.4-fold increase in adjusted relative risk of albuminuria [13]. Conversely, we did not find an association between RRI and albuminuria in our cohort of patients. However, only 42% of our patients were affected by hypertension. Furthermore, obese individuals with no other comorbidities already present higher amounts of albuminuria than the general population, likely reflecting an adverse effect of obesity on kidney function [39]. In severe obesity, as many mechanisms underpin albuminuria, association with RRI might weaken and, in a small cohort like ours, could not be appreciated.

In our study, we found that, in severe obesity, albuminuria is associated with a reduced inhibitory effect of L-NAME on ACh vasodilation. L-NAME blocks the endothelial NO synthase, allowing a direct *ex-vivo* assessment of endothelial function. Very few studies [40] explored the relationship between albuminuria and endothelial function in obesity. Some works reported that their association is already present at very early stages and independently of the glycaemic status [41,42]. However, they did not discriminate between microvascular and macrovascular endothelial function. We here report for the first time that microvascular endothelial dysfunction is related to albuminuria, and, in particular, this association is stronger than the acknowledged one with mean blood pressure [43]. Another peculiarity of our findings is that albuminuria is related to alterations in microvascular function rather than structure. We hypothesize that impaired endothelial function alters glomerular endothelial cell permeability. This is supported by in vivo evidence that the impairment of angiogenesis and endothelial cell permeability, which are related to endothelial dysfunction, occurs very early in the pathogenesis of obesity disease and leads to albuminuria. At the same time, their restoration abrogates the urinary loss of albumin [44,45]. Finally, their different pathophysiological meaning explains the more robust relationship between albuminuria and microvascular endothelial dysfunction rather than blood pressure. Indeed, systemic mean blood pressure is related to a macrovascular- rather than a small vessel-related condition [46]. On the other hand, albuminuria reflects microvascular-only damage, particularly when assessed in a population with a mild-to-moderate cardiometabolic impairment like the one we studied (our participants were free from CV events).

We did not find any association between eGFR and endothelial dysfunction, either. This might also be explained as they are two different and independent factors of cardiometabolic impairment. A similar association has also been reported by Kakutani *et al.*[47], even though they used flow-mediated dilation that is not specific for microvascular endothelial function. However, their findings agree with ours in indicating that while eGFR reflects more of a haemodynamic derangement, albuminuria is more related to a metabolic-specific impairment [19].

In our exploration, the inhibitory effect of L-NAME was not related to systemic blood pressure after adjustment, and we did not observe any difference between normo- and hypertensive patients. However, it is acknowledged that endothelial dysfunction is a feature of essential hypertension that is not related to systemic arterial blood pressure levels [48]. Also, being an early condition in the natural history of cardiometabolic disease, it is possible that the simultaneous presence of obesity, also characterized by microvascular endothelial dysfunction [4], and the possible presence of early compensatory mechanisms [49], hinder the appreciation of a difference between the two groups [50]. Given the above, we would have detected a mild difference between HT and NT. At the same time, we also did not find any difference between microvascular structure between HT and NT. Consistent with what was discussed above, the arterial remodeling seen in our study population might be primarily driven by metabolic factors such as insulin resistance, inflammation and oxidative stress [51], all well represented in severe obesity. These factors may independently contribute to functional and structural arteriolar remodeling independent of the presence of hypertension [52]. Therefore, it is possible that the effect of obesity-specific microvascular dysfunction may have prevailed in the context of severe obesity and concomitant moderately controlled hypertension, masking the hypertension-specific effect on microvascular structure. Although only a limited number of studies have addressed this specific issue, an investigation by Grassi *et al.*[53] further supports the above hypothesis and shows consistency with our findings. In this work, n = 13 patients with obesity (BMI 42.3 ± 1.32 kg/m^2^ and SBP/DBP 133.8 ± 2.8/77.5 ± 1.3 mmHg) vs. *n* = 12 patients with metabolic syndrome and obesity (BMI 45.4 ± 1.5 kg/m^2^ and SBP/DBP 143.5 ± 4.1/91.9 ± 3.8 mmHg; and also higher levels of glucose) had no significant differences in terms of M/L and MCSA (M/L was 0.11 vs. 0.12, *P* = NS and MCSA 22 009.3 μm^2^ vs. 24 760.8 μm^2^, *P* = NS). However, further studies should address this interesting pathophysiological finding.

Our study has some limitations. First, the cross-sectional setting limits the implication of causality and calls for longitudinal studies to address the potential of RRI as a predictor of microvascular remodeling over time and following specific interventions (e.g. bariatric surgery). Similarly, the absence of a control group and of a group of patients with hypertension and without obesity, as well as the unavailability of other hemodynamic parameters, limit the depth of the pathophysiological exploration. Second, the small sample size as well limits the appreciation of effects of smaller magnitude. Third, although an invasive assessment of the microcirculation has been performed, given that the patients included in the present analysis come from different research protocols, the measures of inflammatory markers, endothelial NO bioavailability, and differences in gene expression are consistently available only for a very few numbers of patients to provide a reliable mechanistic exploration. Thus, we do not have included them in the analysis. However, as we stated, we designed our study as a pilot observation to generate hypotheses and promote prospective studies addressing all these relevant clinical, pathophysiological and mechanistic points.

In conclusion, we found that, in patients with severe obesity submitted to bariatric surgery, RRI is related to microvascular remodeling, independent of systemic blood pressure or hypertensive status. These findings suggest that clinical implementation of RRI should be performed in patients with obesity to improve risk stratification. Indeed, obese patients with higher RRI might benefit from more intensive treatment, such as early bariatric surgery, which has proven to rescue microvascular structure and function [36]. Furthermore, our pilot exploration supports the pathophysiologic hypothesis of a tight connection between albuminuria and microvascular function, which deserves to be explored with mechanistic studies aimed at detecting novel and efficient molecular targets for albuminuria (Supplementary S1, Supplemental Digital Content).

## ACKNOWLEDGEMENTS

Author contributions: D.M. and A.M. have made substantial contributions to conception, data collection, statistical analysis and they have been involved in drafting the manuscript. E.D. and F.C. have made contributions to data acquisition. S.T. and M.N. have been involved in revising it critically for important intellectual content. R.M.B., A.V. have made substantial contributions to data interpretation, they have been involved in writing of the manuscript.

Funding: This research did not receive any specific grant from funding agencies in the public, commercial or not-for-profit sectors.

### Conflicts of interest

The authors declare no competing interests. All procedures performed in this study were in accordance with the ethical standards of the institutional and/or national research committee and with the 1964 Helsinki declaration and its later amendments or comparable ethical standards. Informed consent was obtained from all individual participants included in the study.

## Supplementary Material

Supplemental Digital Content
