# Supplementary material for: The renal resistive index is associated with microvascular remodeling in patients with severe obesity
Source: J Hypertens. 2023 Apr 6;41(7):1092–9. doi: 10.1097/HJH.0000000000003434 (PMC10242520; doi:10.1097/HJH.0000000000003434)
Supplement: Supplemental Digital Content [file jhype-41-1092-s001.docx]

|  | **NT** | **HT** | ***p* value** |
| --- | --- | --- | --- |
| n | 8 | 8 |  |
| Age (years) | 55 ± 9 | 55 ± 9 | ns |
| Sex (m/f) | 2/6 | 3/5 | ns |
| Type 2 diabetes (y/n) | 0/8 | 4/4 | 0.02 |
| Albuminuria (y/n) | 1/7 | 1/7 | ns |
| Smoking Habit | 2/6 | 1/7 | ns |
| BMI (kg/m^2^) | 46.4 ± 8.4 | 44.5 ± 3.9 | ns |
| SBP (mmHg) | 128 ± 10 | 131 ± 6 | ns |
| DBP (mmHg) | 82 ± 6 | 84 ± 4 | ns |
| PP (mmHg) | 44 ± 11 | 50 ± 6 | ns |
| MBP (mmHg) | 99 ± 5 | 99 ± 4 | ns |
| Heart Rate (bpm) | 85 ± 11 | 88 ± 12 | ns |
| Glycaemia (mg/dl) | 98 ± 10 | 105 ± 12 | ns |
| HbA1c (mmol/mol) | 38 ± 4 | 46 ± 12 | 0.05 |
| Total Cholesterol (mg/dl) | 201 ± 21 | 182 ± 18 | ns |
| HDL (mg/dl) | 49 ± 14 | 45 ± 10 | ns |
| LDL (mg/dl) | 114 ± 21 | 110 ± 23 | ns |
| Triglycerides (mg/dl) | 122 ± 56 | 126 ± 41 | ns |
| AST (mg/dl) | 27 ± 6 | 27 ± 5 | ns |
| ALT (mg/dl) | 28 ± 6 | 27 ± 9 | ns |
| Uric Acid (mg/dl) | 5.8 ± 1.3 | 6.3 ± 1.4 | ns |
| eGFR (ml/min/1.73m^2^) | 93 ± 17 | 88 ± 24 | ns |
| ACR (mg/g) | 16 [10-20] | 9 [7-15] | ns |
| CRP (mg/dl) | 8.8 ± 4.9 | 9.7 ± 5.1 | ns |

**Suppl. 1. Sensitivity analysis (8 patients NT vs 8 patients HT, matched by age and gender.**

|  | **NT** | **HT** | ***p* value** |
| --- | --- | --- | --- |
| n | 8 | 8 |  |
| Renal resistive index (RRI) | 0.64 ± 0.04 | 0.68 ± 0.03 | 0.049 |
| Renal longitudinal diameter (cm)  Diameter (cm) | 11.4 ± 0.7 | 11.2 ± 0.8 | ns |
| Resting lumen (µm) | 229.9 ± 6.5 | 206.8 ± 7.4 | 0.033 |
| Media thickness (µm) | 21.4 ± 4.6 | 19.4 ± 4.9 | ns |
| M/L ratio | 0.10 ± 0.02 | 0.11 ± 0.01 | ns |
| MCSA (µm^2^) | 15968 ± 3120 | 17280 ± 2955 | ns |
| Maximal vasodilation to Ach (%) | 63.9 ± 7.2 | 62.4 ± 4.1 | ns |
| Maximal vasodilation to Ach+L-NAME (%) | 52.6 ± 5.1 | 50.0 ± 5.4 | ns |
| L-NAME to ACh (%) | 12.2 ± 7.8 | 11.8 ± 5.1 | ns |
